# Borderline Lepromatous Leprosy with Type 1 (Reversal) Reactions in a Chinese Man

**DOI:** 10.4269/ajtmh.14-0491

**Published:** 2015-08-05

**Authors:** Xi’an Fu, Hong Liu, Furen Zhang

**Affiliations:** Shandong Provincial Institute of Dermatology and Venereology, Shandong Academy of Medical Sciences, Shandong, People’s Republic of China; Shandong Provincial Hospital for Skin Diseases, Shandong University, Shandong, People’s Republic of China

**Figure 1.**
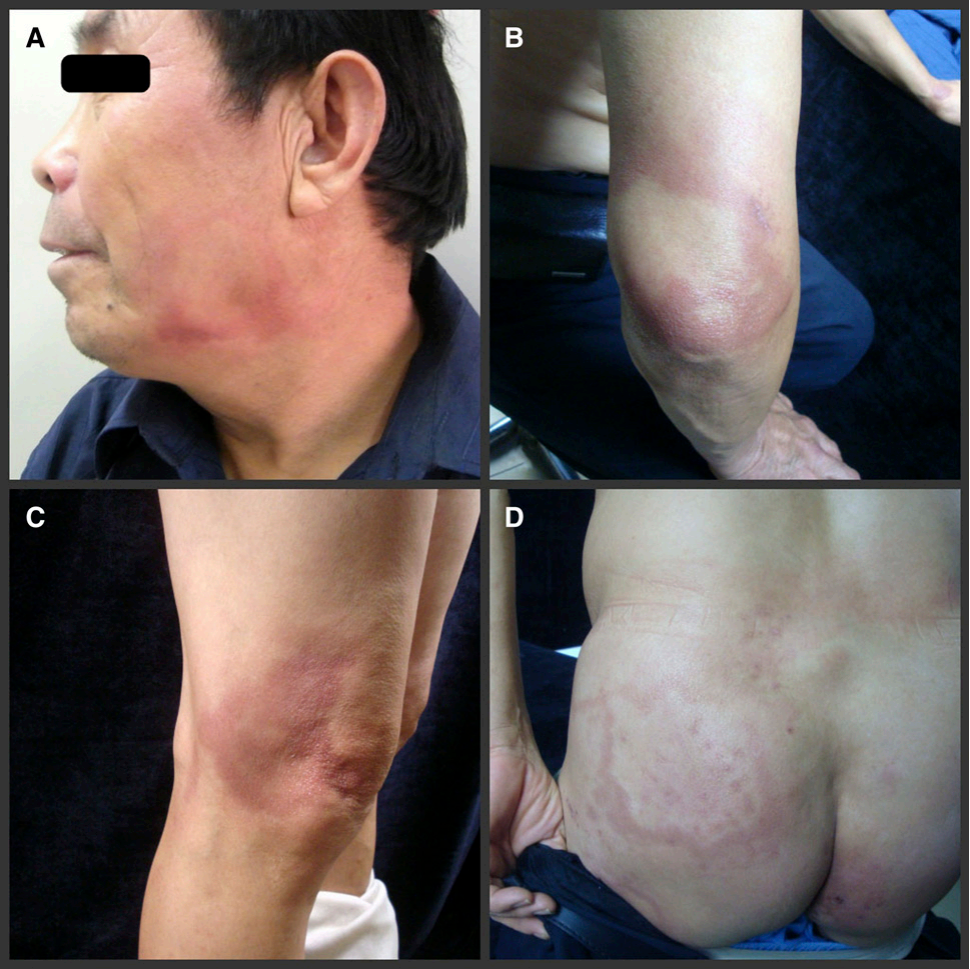
Pre-therapy clinical photograph showing reddish patches and plaques appeared on the patient’s face (**A**), elbow (**B**), knee (**C**), and buttock (**D**).

**Figure 2.**
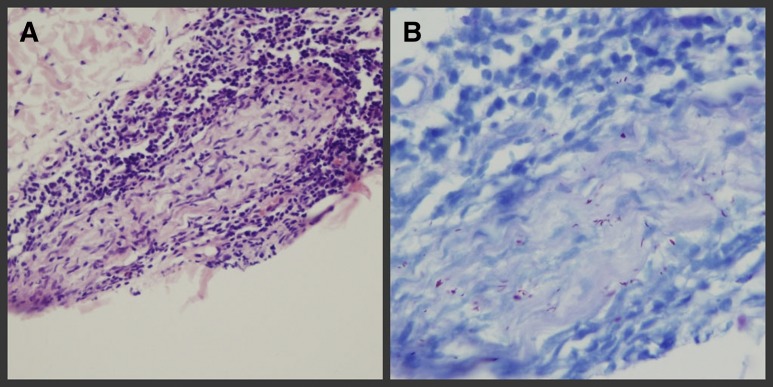
Pre-therapy histopathologic analysis showing (left leg): (**A**) plasmocytic and lymphocytic infiltration surrounding dermal nerve and Schwann cells and inflammatory cell infiltrated into nerve tract (Hematoxylin and Eeosin [H&E] staining ×400), and (**B**) positive staining for lepra bacilli (4+) (acid-fast bacilli [AFB] stain).

**Figure 3.**
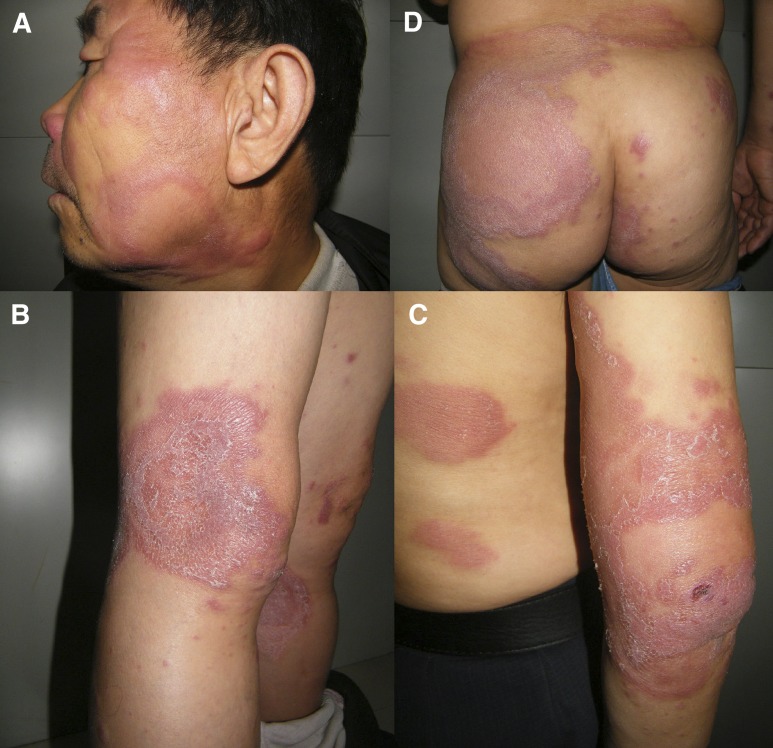
Post-therapy clinical photograph (2 weeks after initiation of multidrug therapy [MDT]). The preexisting lesions in the form of hypochromic macules turned red, edematous, squamous, enlarged, and the inflammatory infiltration aggravated, face (**A**), knee (**B**), elbow (**C**), and buttock (**D**).

**Figure 4.**
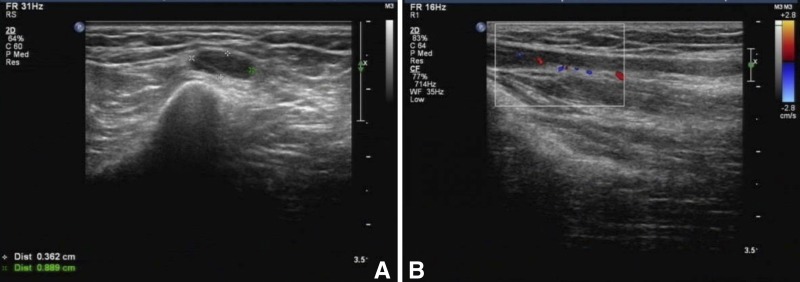
Post-therapy (2 weeks after initiation of multidrug therapy [MDT]) ultrasonography and color Doppler images of peripheral nerve of the patient. (**A**) Cross-section scan of the right side ulnar nerve with hypoechoic fascicles: 0.889 cm at its widest point and (**B**) abnormal blood flow signals around the right ulnar nerve.

A 59-year-old man diagnosed with borderline lepromatous leprosy developed reddish patches and plaques on the face, which progressively enlarged and spread to the trunk and limbs (Figure 1A–D

). Other superficial nerves appeared normal. Biopsy showed plasmocytic and lymphocytic infiltration in the nerve tract, and was 4+ acid-fast bacilli (AFB)–stain positive suggesting *Mycobacterium leprae* (Figure 2A and B

); this was confirmed by real-time polymerase chain reaction (PCR). The HLA-B*13:01 test was negative. Two weeks after rifampin, dapsone, and clofazimine (World Health Organization multidrug therapy [WHO MDT] regimen) were started, the skin lesions (hypochromic macules) became red, edematous, and enlarged (Figure 3A–D

). Both ulnar nerves became tender and thickened; ultrasonography showed reduction of blood flow (Figure 4A and B

). Collectively these findings indicated a type 1 conversion reaction (T1R) (Figure 2C). One year after prednisone was started (40 mg/day for 3 months with progressive tapering), the T1R was found to be completely resolved.

In leprosy, type 1 and type 2 reactions—whether spontaneous or related to treatment—are the main causes of morbidity. T1Rs result from cell-mediated immunity affecting up to 30% of susceptible individuals.^1^ Nonpolar forms of leprosy are the primary risk factor for the occurrence of T1Rs.^2^ Systemic corticosteroids remain the mainstay of treatment of T1Rs.
